# Cross-reactive microbial peptides can modulate HIV-specific CD8^+^ T cell responses

**DOI:** 10.1371/journal.pone.0192098

**Published:** 2018-02-21

**Authors:** Christopher W. Pohlmeyer, Sarah B. Laskey, Sarah E. Beck, Daniel C. Xu, Adam A. Capoferri, Caroline C. Garliss, Megan E. May, Alison Livingston, Walt Lichmira, Richard D. Moore, M. Sue Leffell, Nicholas J. Butler, Jennifer E. Thorne, John A. Flynn, Robert F. Siliciano, Joel N. Blankson

**Affiliations:** 1 Department of Medicine, Johns Hopkins University School of Medicine, Baltimore, Maryland, United States of America; 2 Department of Molecular and Comparative Pathobiology. Johns Hopkins University School of Medicine, Baltimore, Maryland, United States of America; 3 Spondylitis Association of America, Philadelphia, Pennsylvania United States of America; 4 Department of Ophthalmology. Johns Hopkins University School of Medicine, Baltimore, Maryland, United States of America; 5 Howard Hughes Medical Institute, Baltimore, Maryland, United States of America; Jackson Laboratory, UNITED STATES

## Abstract

Heterologous immunity is an important aspect of the adaptive immune response. We hypothesized that this process could modulate the HIV-1-specific CD8^+^ T cell response, which has been shown to play an important role in HIV-1 immunity and control. We found that stimulation of peripheral blood mononuclear cells (PBMCs) from HIV-1-positive subjects with microbial peptides that were cross-reactive with immunodominant HIV-1 epitopes resulted in dramatic expansion of HIV-1-specific CD8^+^ T cells. Interestingly, the TCR repertoire of HIV-1-specific CD8^+^ T cells generated by *ex vivo* stimulation of PBMCs using HIV-1 peptide was different from that of cells stimulated with cross-reactive microbial peptides in some HIV-1-positive subjects. Despite these differences, CD8^+^ T cells stimulated with either HIV-1 or cross-reactive peptides effectively suppressed HIV-1 replication in autologous CD4^+^ T cells. These data suggest that exposure to cross-reactive microbial antigens can modulate HIV-1-specific immunity.

## Introduction

CD8^+^ T cells play a major role in the immune response against HIV-1 infection. The emergence of HIV-specific CTL in primary infection correlates with a drop in viremia to the set point viral load [1,2] and depletion of CD8+ T cells in viremic SIV-infected macaques leads to a significant increase in viral loads [3,4]. Furthermore potent HIV-specific CD8^+^ T cell responses are seen in the majority of subjects who naturally control viral replication (elite suppressors) [5–10]. Heterologous immunity, a key aspect of adaptive immunity, may explain the presence of HIV-specific CD4^+^ T cell responses in HIV-negative subjects [11,12], but this phenomenon has not been as extensively explored in the context of the CD8^+^ T cell response to HIV-1.

We hypothesized that microbial peptides that cross-react with HIV-1 peptides can modulate HIV-1-specific CD8^+^ T cell immunity. We chose to explore this hypothesis in the context of the HLA-B*27 allele, which has been associated with spontaneous control of HIV infection, as well as the HLA-A*02 allele, a common variant with broad clinical relevance. We focused on two epitopes in HIV-1 Gag, KK10 (Gag 263–272, KRWIILGLNK) and SL9 (Gag 77–85, SLYNTVATL), which are immunodominant in HLA-B*27^+^ [13] and HLA-A2^+^ [14] HIV-1 infected individuals, respectively.

We show here that *ex vivo* stimulation with cross-reactive microbial peptides can induce the expansion of CD8^+^ T cells specific for KK10 and SL9. We also demonstrate that in some subjects, the repertoire of CD8^+^ T cells generated by stimulation with HIV-1 peptides is quantitatively distinct from the repertoire of CD8^+^ T cells generated by stimulation with cross-reactive microbial peptides, although both populations of stimulated CD8^+^ T cells are capable of suppressing *ex vivo* HIV-1 infection in autologous CD4^+^ T cells. Together, these data suggest the importance of environmental factors in shaping HIV-1-specific immunity. Characterization of the CD8^+^ T cell response against HIV-1 may inform strategies for a functional or sterilizing HIV-1 cure, many of which implicitly or explicitly depend on CD8^+^ T cell pressure to clear HIV-1 infected cells.

## Materials and methods

### Cross reactive peptide identification

pBLAST search was performed using the BLOSUM62 matrix scoring parameter with a gap cost existence of 10 and gap cost extension of 1. Results from taxid 11676 (HIV), 12721 (Human immunodeficiency virus), 11723 (SIV), 57667 (SHIV), and 32630 (synthetic constructs) were excluded. Additionally, any predicted protein products were excluded. The first 9 results were included in analysis here (KKCR1-KKCR9 and SLCR1-SLCR9).

### Blood donors

All participants provided written, informed consent prior to participation in this study in accordance with Johns Hopkins Medical Institution IRB-approved protocol. Table 1 summarizes characteristics of study participants. Chronic progressors (CP) are HIV-1-positive individuals who began antiretroviral therapy (ART) during chronic infection. All CPs had a viral load of < 20 copies of HIV RNA/mL at the time of this study, with the exception of subject CP2A who was non-adherent to treatment. VC5 is a viremic controller who was started on ART. Elite suppressors (ES) are infected with HIV-1 but have maintained undetectable viral loads without ART. The HLA-B*27^+^, HIV negative subjects were recruited from ankylosing spondylitis and uveitis clinics.

**Table 1:** Characteristics of HIV-infected patients.

Patient	HLA-B27	HLA-A2	cART	Time on suppressive regimens	VL (copies/mL)	CD4 Count (Cells/μL)
CP1A	+		FTC/TAF, DTG	6 years	<20	661
CP2A	+	+	3TC/ABC, DRV/r	NA	16,800	211
CP3A	+		ETV, RAL, DRV/r	6 years	<20	668
CP4A	+		3TC/ABC, DTG	2 years	<20	353
CP11		+	TDF/FTC, DRV/r	8 years	<20	1032
CP12		+	3TC, RAL EFV	3 years	<20	860
CP37		+	AZT/3TC, EFV, ATV/r	8 years	<20	761
CP41		+	3TC/ABC DTG	1 year	<20	948
CP42		+	MVC, RAL, DRV/r	4 years	<20	1140
CP43		+	MVC, RAL, DRV/r	3 years	<20	794
CP44		+	FTC/TDF DRV/r	6 years	<20	692
VC5	+		FTC/TDF DRV/r	2 years	<20	595
ES9	+	+	NA	NA	<20	779
ES31	+		NA	NA	<20	1236

3TC (lamivudine), ABC (abacavir), AZT (zidovudine), FTC (emtricitabine), TAF (tenofovir alafenomide), TDF (tenofovir diproxil), DRV (darunavir), DTG (dolutegravir), EFV (efavirenz) ETV (etravirine), MVC (maravaroc), RAL (raltegravir)

NA: Not applicable. CP2A is non adherent on ART and ES9 and ES31 are not on ART

### Cell culture and peptide stimulation

PBMCs were isolated from fresh whole blood samples by Ficoll density centrifugation. 1x10^6^ PBMCs were stimulated with 1 μg of peptide in 1 mL of RPMI 1640 supplemented with 10% FBS and 10U IL-2 in a 48-well plate for six days. 10U of IL-2 were supplemented every other day. After six days, cells were stained with KK10 or SL9 pentamer and re-stimulated overnight in 1 mL of RPMI 1640 supplemented with 10% FBS, 10U IL-2, 0.5 μg CD28 (CD28.2, BD Biosciences), 0.5 μg CD49d (9F10, BD Biosciences), 3 μM monensin, 1 μg brefeldin A, and 1 μg of peptide. After stimulation for 15 hours, PBMCs were stained with CD3 (UCHT1, Biolegend), CD8 (RPA-T8, Biolegend), IFN-γ (B27, Biolegend), perforin (B-D48, Cell Sciences) and KK10 or SL9 pentamer (Proimmune) before analysis by flow cytometry (BDFACS Canto II) and analysis (FlowJo, TreeStar). Due to availability, samples were run in single replicates.

### IFN-γ ELISpot

Human IFN-γ secretion was measured by stimulation of 2x10^5^ PBMCs in RPMI 1640 supplemented with 10% FBS using precoated ELISpot plates (Mabtech). Processed plates were analyzed by Zellnet Consulting. Samples were run in triplicate wells.

#### TCR deep sequencing

30x10^6^ PBMCs were stimulated with 1 μg/mL peptide and cultured in RPMI 1640 supplemented with 10% FBS and 10U/mL IL-2 for six days. 10U/mL IL-2 was supplemented every other day. CD8^+^ T-cells were isolated by positive magnetic separation (Miltenyi Biotec). CD8^+^ T-cells were stained with CD3 (UCHT1, Biolegend), CD8 (RPA-T8, BD Biosciences), and KK10/SL9 pentamer (Proimmune) prior to sorting CD3^+^/CD8^+^/KK10^+^ or CD3^+^/CD8^+^/SL9^+^ cells (MoFlo XDP, Beckman Coulter). Between 1x10^4^ and 1x10^5^ cells were collected and snap frozen prior to deep sequencing of the CDR3 region of the TCR-β locus (ImmunoSeq, Adaptive Biotechnologies). TCR deep sequence results were exported from the native analysis program and analyzed in Excel. 2 proportion Z test was performed using the analysis tools available in the ImmunoSeq platform.

### PBMC suppression assay

PBMCs were stimulated with 1 μg/mL of peptide in RPMI 1640 supplemented with 10% FBS and 10U/mL IL-2 for five days. 10U/mL IL-2 was supplemented every other day. PBMCs were challenged with 2 μL of 1,000X concentrated IIIB virus (Advanced Biotechnologies), spun at 1,200 x *g* for 15 minutes at 30°C, and cultured for 36 hours. Cells were then stained for CD3 (UCHT1, Biolegend), CD8 (RPA-T8, Biolegend) prior to fixation and permeabilization (Cytofix/Cytoperm, BD Biosciences). Cells were then stained for intracellular Gag (RC57, Beckman Coulter) prior to analysis by flow cytometry (BDFACS Canto II) and analysis (FlowJo, TreeStar). Samples were run in triplicate.

### CD8^+^ T-cell suppression assay

PBMCs were stimulated with 1 μg/mL of peptide in RPMI 1640 supplemented with 10% FBS and 10U/mL IL-2 for 6 days. 10 U/mL IL-2 was supplemented every other day. On day 6, fresh PBMCs were obtained. CD8+ T-cells were isolated from fresh or stimulated PBMCs by positive magnetic separation (Miltenyi Biotec). CD4^+^ T-cells were then isolated from fresh CD8^+^ T-cell-depleted PBMCs and spinoculated with 50ng of pseudotyped NL4-3 virus per 1x10^5^ cells at 1,200 x *g* for 2 hours at 30°C. Infected CD4^+^ T-cells were co-cultured with isolated CD8^+^ T-cells at various effector-to-target ratios for 6, 12, or 24 hours. Cells were stained for CD3 (UCHT1, Biolegend), CD8 (RPA-T8, Biolegend) prior to fixation and permeabilization (Cytofix/Cytoperm, BD Biosciences). Cells were then stained for intracellular Gag (RC57, Beckman Coulter) prior to analysis by flow cytometry (BDFACS Canto II) and analysis (FlowJo, TreeStar). Due to availability, samples were run in single replicates.

### Statistical analysis

Excel was used for analysis of TCR frequency. GraphPad Prism was used for other statistical analysis. Analysis was done using either an ANOVA with p-values adjusted using Holm-Sidak’s multiple comparison test (PBMC suppression test) or a Friedman test with Dunn’s multiple comparison correction (expansion of HIV pentamer-specific CD8^+^ T cells).

## Results

### Cross-reactive microbial peptides stimulate HIV-1-specific CD8^+^ T cells

We first investigated whether cross-reactive microbial peptides could stimulate existing HIV-1-specific CD8^+^ T cells from HIV-1-positive subjects *ex vivo*. Cross-reactive microbial peptide candidates were selected by performing a pBLAST search and limiting results using guidelines from previously described biochemical studies [15,16]; we focused on the anchor residue and considered the P1, P3, and PΩ residues. The pBLAST database is limited to previously characterized proteins and therefore represents only a subset of the sequence diversity found in nature. The microbial peptides we chose are not intended to be comprehensive but are representative of epitopes that could cross-react with HIV-specific T cell receptors (TCRs). Tables 2 and 3 show cross-reactive peptide candidates for KK10 and SL9, respectively.

**Table 2:** Panel of potential KK10 cross-reactive peptides.

Peptide	Sequence	Length	Origin Species	GenBank Accession Number
KK10	KRWIILGLNK	10	*HIV-1*	AIJ50268.1
KK10CR-1	KRLWIILGLIM	11	*Cardiobacterium hominis*	WP_004142625.1
KK10CR-2	ARWIILGLGT	10	*Corynebacterium genitalium*	EFK55173.1
KK10CR-3	QRWIILGLVL	10	*Pseudomonas syringae*	WP_003413168.1
KK10CR-4	KRNTWIILGLYT	12	*Lactobacillus johnsonii*	KRK54456.1
KK10CR-5	KRWIFLGLTI	10	*Imtechella halotolerans*	WP_008237438.1
KK10CR-6	KRWVVLGLTA	10	*Cyanothece sp 7424*	WP_015956625.1
KK10CR-7	QRWIALGLNV	10	*Paenibacillus terrae*	WP_014282856.1
KK10CR-9	QRWIILGLVI	10	*Pseudomonas fragi*	WP_003443111.1
KK10CR-9	ERWAILGLNG	10	*Streptococcus suis*	WP_002942865.1

**Table 3:** Panel of potential SL9 cross-reactive peptides.

Peptide	Sequence	Length	Origin Species	GenBank Accession Number
SL9	SLYNTVATL	9	*HIV-1*	AIJ50268.1
SL9CR-1	SLYNTVVTL	9	*Sediminispirochaeta smaragdinae*	ADK79875.1
SL9CR-2	SLYNTVETL	9	*Syntrophobacter fumaroxidans*	WP_011699462.1
SL9CR-3	DLYDTVATL	9	*Paraburkholderia oxyphila*	WP_028223558.1
SL9CR-4	TLYNTVAAL	9	*Streptomyces sp. S4*	WP_010638463.1
SL9CR-5	SLYDTVAAL	9	*Actinoplanes sp. N902-109*	WP_015620482.1
SL9CR-6	RLYNTVVTL	9	*Candidatus Accumulibacter*	EXI68586.1
SL9CR-7	RLYNIVATL	9	*Neisseria meningitides*	WP_024497022.1
SL9CR-8	ELYNTVNTL	9	*Methanosarcina acetivorans*	WP_011020389.1
SL9CR-9	PLYTTVATL	9	*Bacillus thuringiensis*	AFJ04417.1

**Fig 1:**
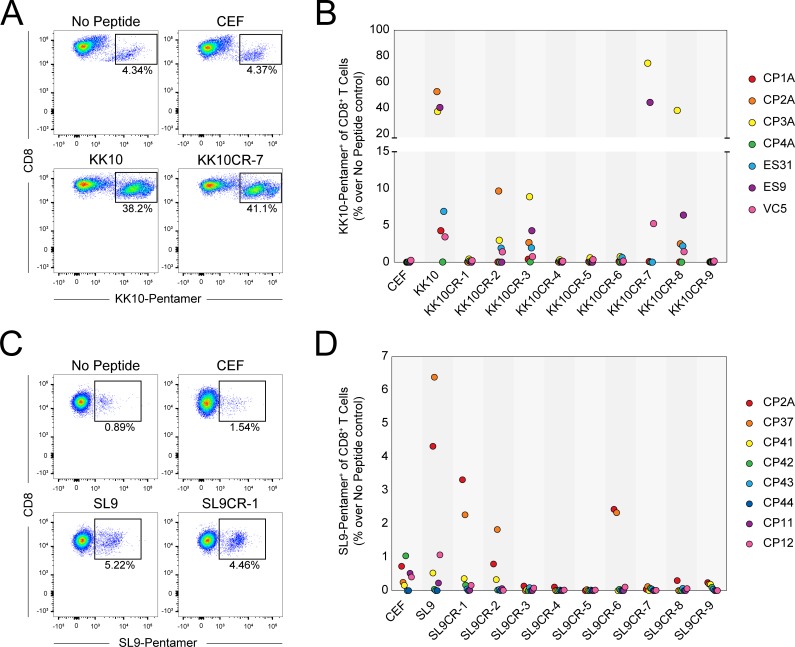
Cross-reactive microbial peptides stimulate HIV-1-specific CD8^+^ T cells. (**A**) Flow cytometric plots of outgrowth of KK10-specific CD8^+^ T cells from PBMCs (subject ES9) after six-day stimulation with no peptide, CEF pooled peptides, KK10 peptide, or KK10CR peptides. (**B**) Summarized results for outgrowth of KK10-specific CD8^+^ T cells from seven HIV-1-positive subject samples stimulated with CEF pooled peptides, KK10 peptide, or individual KK10CR peptides. (**C**) Flow cytometric plots of outgrowth of SL9-specific CD8^+^ T cells from PBMCs (subject CP2A) after six-day stimulation with no peptide, CEF pooled peptides, SL9 peptide, or SL9CR peptides. (**D**) Summarized results for outgrowth of SL9-specific CD8^+^ T cells from eight HIV-positive subject samples stimulated with CEF pooled peptides, SL9 peptide, or individual SL9CR peptides.

To determine whether cross-reactive peptides could expand KK10-specific CD8^+^ T cells, we used KK10 or KK10-cross-reactive (KK10CR) microbial peptides to stimulate PBMCs isolated from HLA-B*27^+^ HIV-1-infected subjects (Table 1) and measured expansion of KK10-specific CD8^+^ T cells by flow cytometry (Fig 1A). In response to KK10 stimulation, we observed expansion of KK10-specific CD8^+^ T cells in six of seven subjects (Fig 1B). The subject who did not respond to KK10 stimulation, a chronic progressor (CP) identified as CP4A, is infected with HIV-1 bearing the R264K escape mutation [17–19] which ablates binding of the KK10 epitope to the HLA-B*27 molecule. Stimulation with four of the nine KK10CR peptides produced responses in at least one subject. Of the six subjects who responded to KK10 stimulation, all six also responded to stimulation with at least one KK10CR peptide. Stimulation with CMV, EBV and influenza (CEF) pooled peptides did not induce outgrowth of KK10-specific CD8^+^ T cells (Fig 1B), indicating that the expansion of KK10-specific cells in response to KK10CR peptide stimulation is not due to nonspecific activation. While there was a significant increase in KK10-specific CD8^+^ T cells after stimulation with KK10 (p = 0.0083 by Friedman test with Dunn’s multiple comparison correction), no other peptide produced significant increases in KK10-specific CD8^+^ T cells after stimulation; this is likely due to the pattern of KK10CR peptide response differed among subjects, suggesting that the TCR repertoire of KK10-specific CD8^+^ T cells varies between individuals and may be shaped by other stimuli in addition to KK10.

A similar pattern of responses was seen in samples from HLA-A2^+^ HIV-1-infected subjects (Table 1) after PBMC stimulation with SL9 or SL9-cross-reactive (SL9CR) microbial peptides (Fig 1C and 1D). Four out of the eight HLA-A2^+^ subjects we tested did not respond to the SL9 peptide at levels above the CEF negative control, likely a reflection of immune escape in this epitope. Due to the high frequency of the HLA-A2 allele in subject populations and the low fitness cost of CD8^+^ T cell escape in the SL9 epitope [20], a large fraction of circulating viruses bear SL9 escape mutations [21]. As a result, subjects may never be exposed to or develop immunity against the wild-type SL9 epitope [22]. There was no significant increase in SL9-specific CD8+ T cells after stimulation with SL9 peptide, due to the heterogeneity of recognition of SL9.

### Characterization of CD8^+^ T-cell response to KK10CR peptides

**Fig 2:**
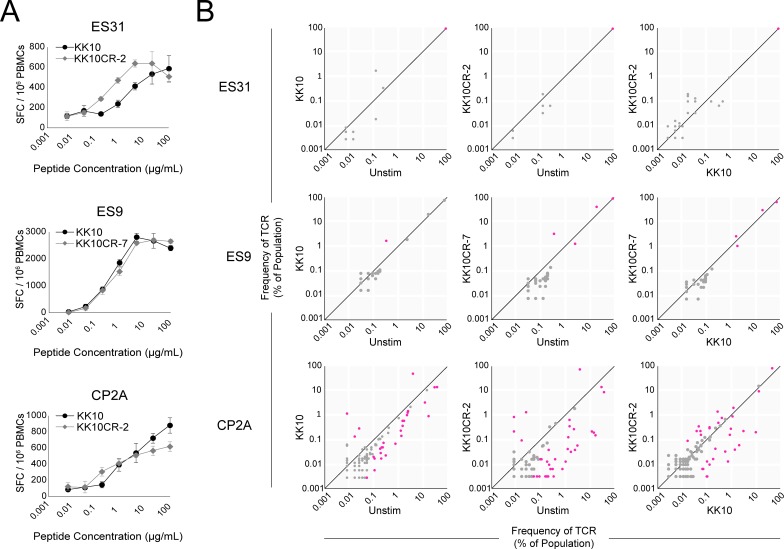
KK10 and KK10-cross-reactive microbial peptides differentially expand KK10-specific CD8^+^ T cells. (**A**) IFN-y release by ELISpot from HIV-positive HLA-B*27^+^ subject PBMCs stimulated with dilutions of KK10 or KK10CR peptides. (**B**) Characterization of KK10-specific TCR repertoires after PBMC stimulation with KK10 or KK10CR peptides. PBMCs from HIV-1-positive HLA-B*27^+^ subjects were stimulated with peptides for six days, and KK10-specific CD8^+^ T cells were sorted from fresh (unstimulated), KK10-stimulated, or KK10CR-stimulated PBMCs. Frequencies of unique TCR clones as measured by diversity in the TCR-β CDR3 region were quantified by ImmunoSEQ deep sequencing. Each dot shows the frequency of a single TCR clone in the specified KK10-specific CD8^+^ T cell populations. TCR clones that have the same frequency in both populations compared on a plot fall on the diagonal line. Magenta dots indicate TCR clones that are present at significantly different frequencies (minimum 20 reads in either condition, p<0.005, Fisher’s Exact test with Benjamini-Hochberg correction. S1 Table shows all associated p-values).

We next asked whether KK10 or KK10CR peptide stimulations induce qualitatively similar *ex vivo* responses in HIV-1-positive HLA-B*27^+^ subject samples. To compare the functional avidities of KK10 and KK10CR microbial peptides, we stimulated PBMCs with peptide and measured IFN-γ release by ELISpot (Fig 2A). In all three subjects tested, stimulation with KK10CR microbial peptides induced IFN-γ-secreting cells at frequencies similar to those induced by stimulation with KK10 peptide.

### Characterization of CD8^+^ T cell response to HIV-1 and cross-reactive peptides

We next asked whether HIV-1 and cross-reactive peptide stimulations induce similar *ex vivo* responses in HIV-1-positive subject samples. To characterize the HIV-1-specific CD8^+^ TCR repertoires induced by stimulation with HIV-1 peptides or cross-reactive microbial peptides, we stimulated PBMCs from HIV-1-positive subjects with either HIV-1 or cross-reactive microbial peptides, sorted HIV-1 peptide-specific CD8^+^ T cells, and used immunoSEQ deep sequencing to characterize their TCR repertoires. Individual TCRs were identified by sequencing the highly variable CDR3 region of the TCR-β gene. We used this protocol to assess the KK10-specific repertoire in HLA-B*27^+^ subjects and the SL9-specific CD8^+^ T cell repertoire in HLA-A2^+^ subjects.

**Fig 3:**
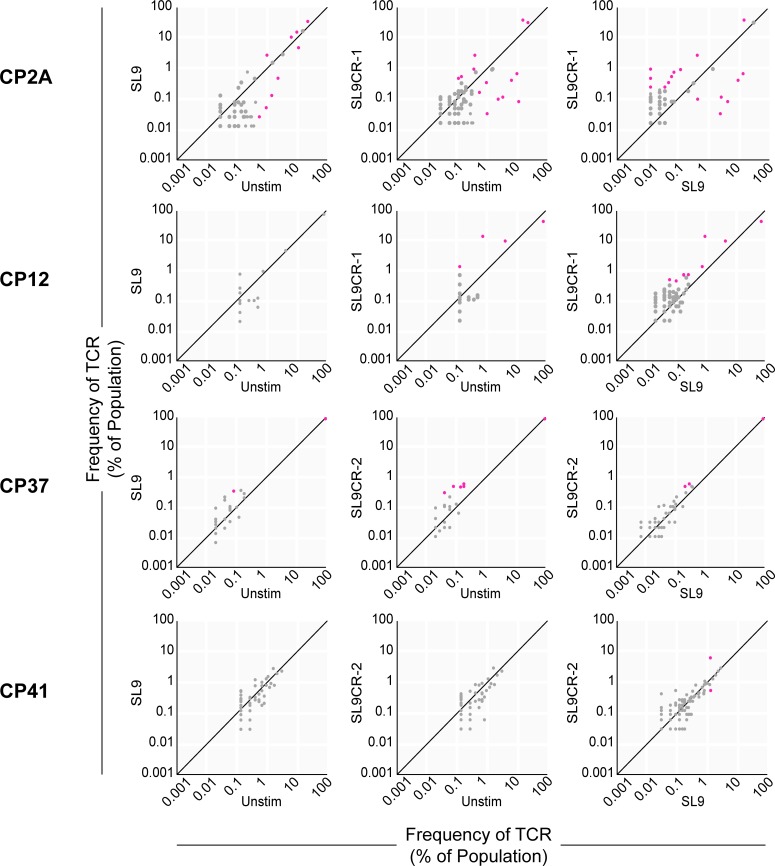
SL9 and SL9-cross-reactive microbial peptides differentially expand SL9-specific CD8^+^ T cells. Characterization of SL9-specific TCR repertoires after PBMC stimulation with SL9 or SL9CR peptides. PBMCs from HIV-1-positive HLA-A2^+^ subjects were stimulated with peptides for six days, and SL9-specific CD8^+^ T cells were sorted from fresh (unstimulated), SL9-stimulated, or SL9CR-stimulated PBMCs. Frequencies of unique TCR clones as measured by diversity in the TCR-β CDR3 region were quantified by ImmunoSEQ deep sequencing. Each dot shows the frequency of a single TCR clone in the specified SL9-specific CD8^+^ T cell populations. TCR clones that have the same frequency in both populations compared on a plot fall on the diagonal line. Magenta dots indicate TCR clones that are present at significantly different frequencies (minimum 20 reads in either condition, p<0.005, Fisher’s Exact test with Benjamini-Hochberg correction. S2 Table shows all associated p-values).

In all three HLA-B*27^+^ subjects tested and three of the four HLA-A2^+^ subjects tested, there were statistically significant differences between the frequencies HIV-specific TCR repertoires of stimulated and unstimulated CD8^+^ T cells (S1 Table and S2 Table). However, the number of TCR clones that were significantly different across the different stimulation conditions varied by subject. In subjects ES31 (HLA-B*27^+^, Fig 2B) and CP37 (HLA-A2^+^, Fig 3), a single TCR clone predominated in all three conditions shown; the difference in the frequency of the predominant TCR in the different stimulation conditions, while significant, likely would not have substantial difference in functional activity. In subjects ES9 and CP2A, the TCR repertoire of HIV-1-specific CD8^+^ T cells changed considerably in response to stimulation with HIV-1 and cross-reactive peptides with respect to the TCR repertoire of unstimulated HIV-1 peptide-specific CD8^+^ T cells. Interestingly, in CP2A, who is positive for both HLA-A2 and HLA-B*27 and was viremic at the time of sample collection (Table 1), stimulation with HIV-1 peptides or with cross-reactive peptides resulted in the expansion of distinct HIV-1 peptide-specific TCR repertoires, for both KK10 (Fig 2B) and SL9 (Fig 3).

### Suppression of HIV replication after stimulation with HIV-1 or cross-reactive peptides

CD8^+^ T cells are much more effective at controlling HIV-1 replication after stimulation with HIV-infected cells or peptides [8]. Based on this concept, we developed a novel PBMC suppression assay to measure inhibition of HIV-1 replication by peptide-stimulated cells. Unfractionated PBMCs from HIV-infected subjects were either left unstimulated (as a negative control) or were stimulated with HIV-1 peptides or cross-reactive microbial peptides for five days. After stimulation, we infected the unfractionated PBMCs with concentrated IIIB virus and incubated for 40 hours, then quantified the percentage of infected CD4^+^ T cells by staining for intracellular HIV-1 Gag. Suppression of 20% or less was defined as below the limit of quantification, defined by responses observed in HIV-negative healthy subjects (data not shown).

**Fig 4:**
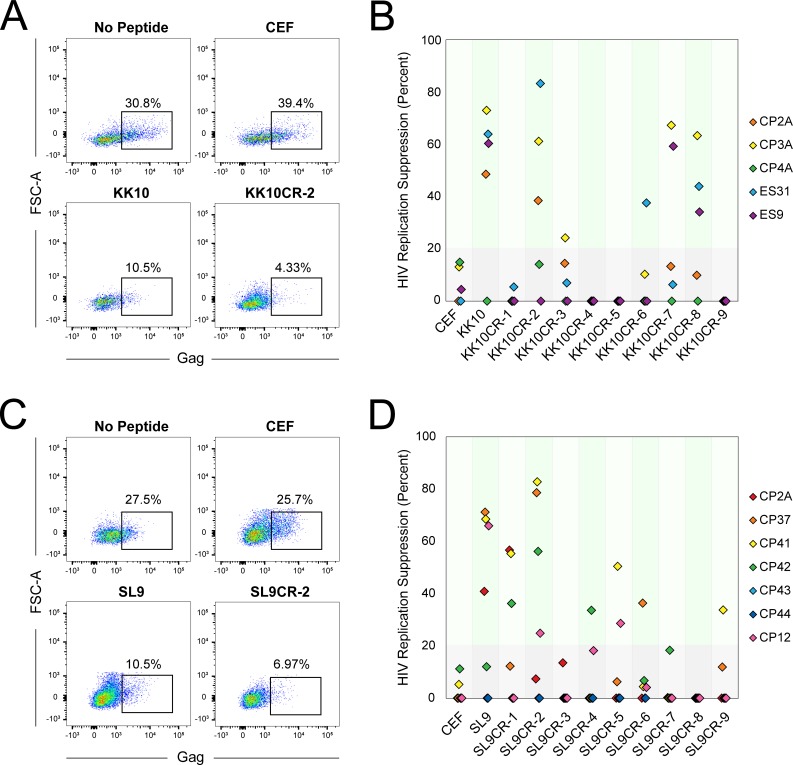
PBMCs stimulated with cross-reactive microbial peptides can suppress HIV-1 infection. (**A-B**) Results of PBMC suppression assay. PBMCs from HIV-1-positive HLA-B*27^+^ subjects were stimulated for five days with no peptide, CEF pooled peptides, KK10 peptide, or KK10CR peptide and then infected with highly concentrated IIIB virus. After 40 hours, infection of CD3^+^/CD8^-^ cells was measured by staining for intracellular HIV-1 Gag. Values below 20% (in grey) fall below the limit of quantification. Part **A** shows flow cytometric results for subject ES31; part **B** shows summarized results for five subjects with CEF pooled peptides, KK10 peptide, and individual KK10CR peptides (**C-D**) PBMCs from HIV-1-positive HLA-A2^+^ subjects were stimulated for five days with no peptide, CEF pooled peptides, SL9 peptide, or SL9CR peptide and then infected with highly concentrated IIIB virus. After 40 hours, infection of CD3^+^/CD8^-^ cells was measured by staining for intracellular HIV-1 Gag. Part **C** shows flow cytometric results for subject CP37; part **D** shows summarized results for seven subjects with CEF pooled peptides, SL9 peptide, and individual SL9CR peptides.

In both HLA-B*27^+^ (Fig 4A and 4B) and HLA-A2^+^ (Fig 4C and 4D) subject samples, stimulation of PBMCs with HIV-1 or cross-reactive microbial peptides resulted in much lower frequencies of infected target cells compared to unstimulated PBMCs, whereas stimulation with negative control CEF peptides had no effect on the frequency of infected cells. The results of this suppression assay are summarized in Fig 4B (KK10) and 4d (SL9), and p-values for individual subjects are summarized in S3 Table. Overall, the magnitude of suppression in cross-reactive peptide-stimulated cultures reached a similar magnitude to the magnitude of suppression in cultures stimulated with HIV-1 peptides.

### Purified T cell suppression assay

**Fig 5:**
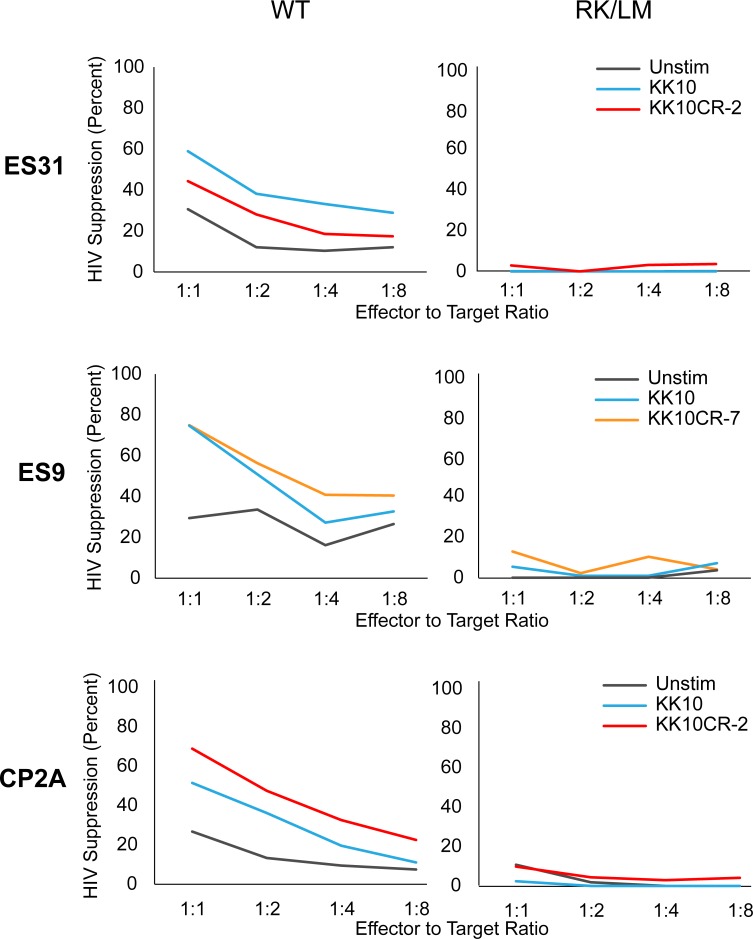
CD8^+^ T cells stimulated with cross-reactive microbial peptides can suppress HIV-1 infection. Purified CD4^+^ T cells from three HIV-1-positive HLA-B*27^+^ subjects were spinoculated with single-round NL4-3 GFP reporter virus and then co-cultured with peptide-stimulated or unstimulated (fresh *ex vivo*) autologous CD8^+^ T cells. Viral suppression was measured at four different effector-to-target cell ratios. Left panels show suppression of wild-type (WT) NL4-3 reporter virus, and right panels show suppression of NL4-3 harboring the R264K and L268 mutations in KK10, which prevent KK10 presentation on HLA-B*27 MHC molecules.

To confirm that the suppression of viral replication seen in the PBMC suppression assay was mediated by HIV-1-specific effector CD8^+^ T cells, we performed a variation of the assay using purified cells. We isolated and infected CD4^+^ T cells, and then cultured them alone or in the presence of autologous purified CD8^+^ T cells. In this purified cell suppression assay, CD8^+^ T cells stimulated with KK10 and KK10CR peptides suppressed viral replication more effectively than unstimulated CD8^+^ T cells (Fig 5). This suppression was abrogated when the target CD4^+^ T cells were infected with an HIV-1 clone harboring R264K/L268M escape mutations.

The purified cell suppression assay confirms that stimulation with HIV-1 or cross-reactive peptides enhance the ability of CD8^+^ T cells to suppress HIV-1 infection. Importantly, the magnitude of suppression is comparable between KK10 and KK10CR peptides, demonstrating again the potential importance of cross-reactive antigens as a modulator of HIV-1 specific immunity.

Most importantly, the results of the purified cell suppression assay highlight the key role that CD8^+^ T cells play in HIV-1 immunity. We saw a dose-dependent relationship between effector-to-target cell ratio and suppression of HIV-1 replication (Fig 5), indicating that the CD8^+^ T cells are directly responsible for suppressing infection in autologous CD4^+^ T cells. We also saw that this suppression depends on CD8+ T cells specifically targeting the KK10 epitope, regardless of whether the CD8^+^ T cells were stimulated with KK10 or KK10CR peptides.

### KK10 responses in HIV-negative HLA-B*27^+^ donors

**Fig 6:**
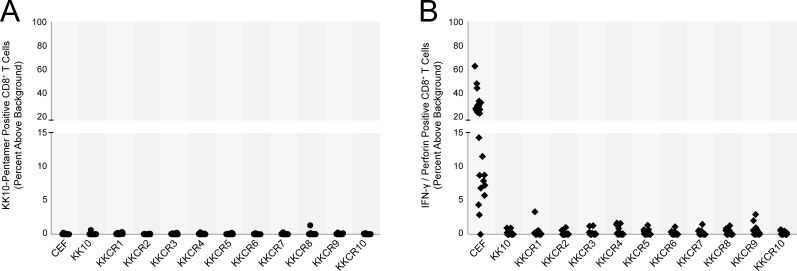
Cross-reactive microbial peptides do not expand HIV-1-specific CD8^+^ T cells in HIV-negative donors. 25 HIV-negative HLA-B*27^+^ donors were screened for the presence of KK10-specific CD8^+^ T cells. Donor PBMCs were stimulated with KK10 peptide, KK10CR peptides or negative control CEF pooled peptides for six days, and the percentage of (**A**) KK10-specific and (**B**) IFN-γ^+^Perforin^+^ CD8^+^ T cells was quantified by flow cytometry.

We next asked whether we could detect HIV-specific immune responses in HIV-negative subjects possibly as a result of exposure to cross reactive epitopes. Although HIV-specific CD4^+^ T-cells have been observed in HIV-seronegative individuals [11,12], we were not able to detect KK10-specific CD8^+^ T-cells in PBMCs isolated from 25 HLA-B*27^+^ HIV-seronegative individuals upon stimulation with KK10 peptide by either staining with a pentamer (Fig 6A) or with antibodies to IFN-γ and perforin (Fig 6B). In contrast we were able to detect CD8^+^ T cell responses to CEF peptides (Fig 6B).

## Discussion

Recent studies have suggested that cross reactive microbial peptides can induce HIV-specific CD4^+^ T cell response in HIV-negative subjects [11,12]. In contrast, there is less known about the role of heterologous immunity in the induction and maintenance of HIV-specific CD8^+^ T cell responses. Several studies have shown that cross reactivity between the HIV-1 SL9 peptide and the immunodominant influenza peptide GL9 [23–24] or the HCV peptide AL9 [25] can occur in HIV-infected HLA-A2^+^ subjects. Additionally, several studies have looked at the ability of the TCR to cross-react to escape variants within HIV epitopes [26–30]. However, it is not known whether similar responses are seen in subjects with other HLA alleles and whether cross reactivity to other microbial peptides occurs.

In this study we looked first at HLA-B*27^+^ subjects since responses to a single immunodominant epitope is associated with control of viral replication [18,19]. We then looked at HLA-A2^+^ subjects given the high prevalence of this allele. We found significant cross reactivity between several microbial peptides and the KK10 and SL9. We show here that these cross-reactive microbial antigens can stimulate and expand HIV-specific CD8^+^ T cell responses. We also show significant differences between the TCR repertoires of CD8^+^ T cells expanded with HIV-1 versus cross-reactive microbial peptides. Interestingly, the pattern of response to cross-reactive microbial peptides differed among subjects, supporting the conclusion that the TCR repertoire of HIV-specific CD8^+^ T cells may be shaped by other stimuli in addition to HIV-1 infection. These results suggest that in some subjects, microbial peptides can modulate HIV-specific immunity by differentially stimulating different HIV-specific TCRs. This finding, which is consistent in subjects with both protective and non-protective HLA alleles, may have implications for the recognition of polymorphisms within HIV-1 epitopes [26,27].

We have also shown that stimulation with cross-reactive peptides can enhance the ability of HIV-specific CD8^+^ T cells to control HIV-1 replication. Together, the data from two suppression assays show that cross-reactive peptide-stimulated CD8^+^ T cells can suppress HIV-1 infection. Additionally, we show that suppression by KK10CR-stimulated CD8^+^ T cells relies on a KK10 epitope-dependent mechanism.

Interestingly, we did not see KK10-specific CD8^+^ T cell responses in HIV-negative HLA-B*27^+^ subjects. In a similar study, SL9-specific CD8^+^ T cell responses were not seen in HIV-negative HLA-A2^+^ positive subjects [24]. It’s not clear why *de novo* HIV-specific CD4^+^ T cell responses would be easier to induce by cross reactive peptides than HIV-specific CD8^+^ T cell responses, but our data do suggest that cross reactive microbes can stimulate the robust expansion of pre-existing HIV-specific CD8^+^ T cell responses in some subjects. Taken together, the results presented here demonstrate that cross-reactive microbial peptides can modulate HIV-specific CD8^+^ T cell responses. These findings could inform vaccine development and the design of CD8^+^ T cell-based immunotherapy for eradication studies.

## Supporting information

S1 Table TCR information from HLA-B*27 KK10-specific CD8+ TCR deep sequencing. (DOCX)

S2 Table TCR information from HLA-A*02 SL9-specific CD8+ TCR deep sequencing. (DOCX)

S3 Table Individual p-values from PBMC suppression assay. (DOCX)
