# Supplementary material for: Cross-reactive microbial peptides can modulate HIV-specific CD8+ T cell responses
Source: PLoS One. 2018 Feb 21;13(2):e0192098. doi: 10.1371/journal.pone.0192098 (PMC5821448; doi:10.1371/journal.pone.0192098)
Supplement: S1 Table — (DOCX) [file pone.0192098.s001.docx]

**S1 Table. TCR information from HLA-B*27 KK10-specific CD8^+^ TCR deep sequencing**

| ES31 | Frequency (%) | | | p-value | | |
| --- | --- | --- | --- | --- | --- | --- |
| TCR Sequence | Unstimulated (n=788) | KK10  (n=5,519) | KK10CR-2 (n=3,109) | Unstimulated vs KK10 | Unstimulated vs KK10CR-2 | KK10 vs KK10CR-2 |
| CSAADRDSSGSPYGYTF | 93.7817 | 94.7092 | 94.6285 | 2.01E-08 | 2.42E-03 | 1.92E-07 |
|  |  |  |  |  |  |  |
|  |  |  |  |  |  |  |
| ES9 | Frequency (%) | | | p-value | | |
| TCR Sequence | Unstimulated (n=3,399) | KK10  (n=6,637) | KK10CR-7 (n=14,339) | Unstimulated vs KK10 | Unstimulated vs KK10CR-7 | KK10  vs K10CR-7 |
| CASSFDSNEQFF | 16.9167 | 19.5420 | 29.9672 | *n/s* | 3.25E-56 | 6.88E-59 |
| CASSLDRQPQHF | 0.323625 | 1.61217 | 2.55248 | 7.90E-10 | 1.17E-21 | 1.29E-05 |
| CASSLDSYEQYF* | 2.29479 | 1.82311 | 1.02518 | *n/s* | 2.80E-08 | 3.25E-06 |
| CASSLDSYEQYF* | 76.1106 | 73.7231 | 64.0003 | *n/s* | 7.48E-47 | 2.09E-46 |
|  |  |  |  |  |  |  |
|  |  |  |  |  |  |  |
| CP2A | Frequency (%) | | | p-value | | |
| TCR Sequence | Unstimulated (n=13,450) | KK10 (n=36,697) | KK10CR-2 (n=32,825) | Unstimulated vs KK10 | Unstimulated vs KK10CR-2 | KK10 vs KK10CR-2 |
| CARSQTSGRAGGASDTQYF | 0.804615 | 0.728115 | 0.012318 | *n/s* | 6.93E-52 | 2.80E-67 |
| CASAGTTGELFF | 0 | 0.057957 | 0.144731 | 2.21E-03 | 1.40E-07 | 3.57E-04 |
| CASASGRVNNEQFF | 1.29447 | 0.563007 | 0.030794 | 3.88E-15 | 6.64E-79 | 4.67E-44 |
| CASKQASGDYEQYF | 0.272521 | 0 | 0.003079 | 2.23E-21 | 9.58E-19 | *n/s* |
| CASRLDRNEQYF | 0.204391 | 0.071756 | 0.006159 | 2.41E-04 | 5.57E-13 | 9.31E-06 |
| CASSADRDEQYF | 2.33794 | 2.07127 | 0.055429 | *n/s* | 1.89E-141 | 1.27E-180 |
| CASSARTGELFF | 0 | 0.226307 | 0.270986 | 1.20E-11 | 1.19E-13 | *n/s* |
| CASSGKTGELFF | 0 | 0.016548 | 0.083143 | *n/s* | 1.53E-04 | 6.84E-05 |
| CASSGSTGELFF | 0.022710 | 0.275984 | 1.24407 | 1.73E-10 | 3.69E-55 | 2.09E-52 |
| CASSHKTGELFF | 0 | 0.063476 | 0.086223 | 1.38E-03 | 9.37E-05 | *n/s* |
| CASSLGVLDYGYTF | 25.7078 | 13.1865 | 8.14498 | 8.78E-225 | 0 | 4.20E-102 |
| CASSLQGEDYGYTF | 0.007591 | 0.055160 | 0.206319 | *n/s* | 3.40E-09 | 1.81E-08 |
| CASSLSLLAGSLISSYNEQFF | 0.189251 | 0.046917 | 0.316670 | 9.91E-06 | *n/s* | 1.39E-18 |
| CASSPGAFSYEQYF | 1.70326 | 0.957664 | 0.255589 | 4.05E-11 | 3.88E-58 | 8.32E-34 |
| CASSPGLFSYEQYF | 0.151400 | 0.041398 | 0.070069 | 1.59E-04 | *n/s* | *n/s* |
| CASSPGQFGNTIYF | 0.968963 | 0.223547 | 0.040032 | 1.14E-25 | 5.44E-54 | 1.05E-11 |
| CASSPGQLGNEQFF* | 1.02195 | 0.137992 | 0.120096 | 2.93E-39 | 2.13E-40 | *n/s* |
| CASSPGQLGNEQFF* | 0.319703 | 0.295107 | 0.439648 | *n/s* | *n/s* | 1.83E-03 |
| CASSPGQLGNTIYF* | 1.13550 | 0.563007 | 0.609719 | 2.28E-10 | 1.33E-08 | *n/s* |
| CASSPGQLGNTIYF* | 0.174111 | 0.046917 | 0.036953 | 4.30E-05 | 6.69E-06 | *n/s* |
| CASSPGQLGNTIYF* | 0.015140 | 0.129712 | 0.088347 | 6.66E-05 | *n/s* | *n/s* |
| CASSPGQTGNTIYF | 1.86223 | 1.38820 | 0.036953 | 1.78E-04 | 8.48E-116 | 3.86E-121 |
| CASSPGQYGNTIYF* | 20.4164 | 12.9961 | 13.1397 | 2.43E-88 | 7.02E-83 | *n/s* |
| CASSPGQYGNTIYF* | 10.2930 | 9.91505 | 0.184763 | *n/s* | 0 | 0 |
| CASSPGRLGNTIYF | 1.14307 | 0.350500 | 0.335653 | 2.28E-22 | 8.70E-23 | *n/s* |
| CASSPGRLSNEQYF | 0.182177 | 0.129627 | 0.009238 | *n/s* | 1.22E-10 | 2.08E-10 |
| CASSPGVFGNTIYF | 8.59198 | 3.11034 | 0.206319 | 1.21E-130 | 0 | 2.94E-228 |
| CASSQGERSNEQFF | 0.141264 | 0.104804 | 0.003074 | *n/s* | *n/s* | 7.07E-10 |
| CASSQGLFGNEQFF | 0.126394 | 0.099288 | 0.003074 | *n/s* | *n/s* | 2.54E-09 |
| CASSQGLFSNEQFF | 11.9076 | 0.869349 | 0.144731 | 0 | 0 | 3.71E-44 |
| CASSQGLFSSEQYF | 0.598032 | 0.066236 | 0.006159 | 5.23E-26 | 3.25E-40 | 3.08E-05 |
| CASSQGMFGNEQFF | 0.311219 | 0.182029 | 0.015397 | *n/s* | 1.88E-17 | 1.70E-13 |
| CASSQGQLGNTIYF | 1.80167 | 1.21433 | 1.75552 | 1.42E-06 | *n/s* | 3.99E-09 |
| CASSQGQLSTEAFF | 0.158970 | 0.027598 | 0.003079 | 2.15E-06 | 7.31E-11 | *n/s* |
| CASSQGQWSTEAFF | 0.249811 | 0.022079 | 0.006159 | 1.03E-12 | 4.65E-16 | *n/s* |
| CASSQGRTGAEQYF | 0.151814 | 0.102046 | 0.012318 | *n/s* | 4.69E-08 | 3.59E-07 |
| CASSQGVASAEQYF | 0.096654 | 0.063434 | 0 | *n/s* | *n/s* | 4.33E-07 |
| CASSQRTGELFF* | 0 | 0.129712 | 0.292542 | 9.56E-07 | 1.71E-14 | 2.89E-06 |
| CASSQRTGELFF* | 0 | 0.082795 | 0.113937 | 1.17E-04 | 4.57E-06 | *n/s* |
| CASSQSTGELFF | 0.007570 | 1.10946 | 0.797561 | 4.65E-53 | 3.66E-37 | 2.72E-05 |
| CASSQTTGELFF | 0 | 0.038612 | 0.206319 | *n/s* | 2.14E-10 | 6.67E-11 |
| CASSRRTGELFF | 2.90689 | 46.0534 | 68.4363 | 0 | 0 | 0 |
| CASSSRTGELFF | 0 | 0.408456 | 0.692862 | 1.71E-20 | 9.25E-34 | 5.21E-07 |
| CASSTRTGELFF | 0 | 0.129712 | 0.267907 | 9.56E-07 | 1.92E-13 | 4.20E-05 |
| CASSYGIGPERGKTQYF | 0.166540 | 0.016559 | 0.003079 | 1.56E-08 | 2.20E-11 | *n/s* |

* identical TCR sequence by AA but differs by nucleic acid sequence.

*n/s* indicates no significant difference or too few cells to make comparison
