# Supplementary material for: Cross-reactive microbial peptides can modulate HIV-specific CD8+ T cell responses
Source: PLoS One. 2018 Feb 21;13(2):e0192098. doi: 10.1371/journal.pone.0192098 (PMC5821448; doi:10.1371/journal.pone.0192098)
Supplement: S2 Table — (DOCX) [file pone.0192098.s002.docx]

**S2 Table. TCR information from HLA-A*02 SL9-specific CD8^+^ TCR deep sequencing**

| CP2A | Frequency (%) | | | p-value | | |
| --- | --- | --- | --- | --- | --- | --- |
| TCR Sequence | Unstimulated (n=3,612) | SL9 (n=7,730) | SL9CR-1 (n=6,102) | Unstimulated vs SL9 | Unstimulated vs SL9CR-1 | SL9  vs SL9CR-1 |
| CASSDTVGYEQYF | 13.3905 | 5.092210 | 0.096432 | 1.06E-43 | 7.78E-173 | 2.14E-79 |
| CASSFDSEAFF | 0.456026 | 0.495595 | 3.20154 | *n/s* | 8.45E-20 | 8.63E-33 |
| CASSGTKSYEQYF | 2.77045 | 0.509221 | 0.115718419 | 7.70E-20 | 1.69E-29 | 1.42E-04 |
| CASSHDRLQYF | 1.18734 | 2.903936141 | 0.038573 | 4.01E-08 | 8.70E-14 | 1.95E-46 |
| CASSHGGRNEQFF | 11.6755 | 16.5841 | 0.790743 | 1.22E-10 | 3.72E-110 | 3.64E-237 |
| CASSHLPPGFANTGELFF | 0.130293 | 0.013767 | 0.559306 | *n/s* | 1.90E-03 | 1.38E-10 |
| CASSLDSEQYF | 18.1759 | 17.9653 | 45.4966 | *n/s* | 7.29E-147 | 1.69E-246 |
| CASSLGVLDYGYTF | 1.12137 | 0.055051 | 0.405014 | 1.50E-14 | 2.24E-04 | 1.69E-05 |
| CASSLSPSGRSGELFF | 0 | 0.013767 | 0.291886 | *n/s* | 1.82E-03 | 1.76E-05 |
| CASSPATGGPREQFF | 0.221439 | 0.812225 | 0 | *n/s* | *n/s* | 2.68E-14 |
| CASSPAWWEGAYEQYF | 0.423453 | 0.013767 | 1.11861 | *n/s* | 7.58E-04 | 1.86E-21 |
| CASSPGQYGNTIYF | 0.659631 | 0.027525 | 0.192864 | 2.75E-09 | 1.13E-03 | *n/s* |
| CASSPTGATGANVLTF | 0.415282 | 0.082599 | 0.895116 | *n/s* | *n/s* | 2.81E-12 |
| CASSQTIYGYTF | 0.162866 | 0.055066 | 0.636451 | *n/s* | 1.99E-03 | 1.90E-09 |
| CASSRRTGELFF | 1.71504 | 0.137627 | 1.08971 | 6.68E-19 | *n/s* | 3.92E-13 |
| CASSSTKSYEQYF | 7.68470 | 11.3130 | 0.482160 | 1.65E-08 | 1.89E-73 | 1.68E-161 |
| CATQGTSGGADTQYF | 27.2098 | 37.2832 | 37.7049 | 3.37E-23 | 3.78E-24 | *n/s* |
| CATSGTLAGGGETQYF | 3.90879 | 3.11123 | 0.135005 | *n/s* | 2.51E-43 | 2.39E-43 |
|  |  |  |  |  |  |  |
|  |  |  |  |  |  |  |
| CP12 | Frequency (%) | | | p-value | | |
| TCR Sequence | Unstimulated (n=765) | SL9 (n=4,871) | SL9CR-1 (n=4,539) | Unstimulated vs SL9 | Unstimulated vs SL9CR-1 | SL9 vs  SL9CR-1 |
| CASNSGNTIYF | 0 | 0.102648 | 0.462657 | *n/s* | *n/s* | 5.19E-04 |
| CASSGPTEHSPLHF | 0 | 0.061589 | 0.506720 | *n/s* | *n/s* | 1.12E-05 |
| CASSHVGNTEAFF | 4.44444 | 4.76288 | 9.91408 | *n/s* | 1.82E-10 | 5.27E-27 |
| CASSLEDQPQHF | 0.784314 | 0.964894 | 14.1881 | *n/s* | 2.93E-45 | 1.38E-167 |
| CASSLGVRSGYEQFF | 0 | 0.184767 | 0.749064 | *n/s* | *n/s* | 1.80E-05 |
| CASSQDPGLDYYGYTF | 85.6209 | 77.4584 | 44.6134 | *n/s* | 9.55E-83 | 4.56E-240 |
| CASSVDRGPSGGELFF | 0.130719 | 0.184767 | 0.727032 | *n/s* | *n/s* | 3.03E-05 |
| CASSYSWGATNTEAFF | 0 | 0.266886 | 0.749064 | *n/s* | *n/s* | 3.55E-04 |
| CASTAGRGQPQHF | 0.130719 | 0.780127 | 1.38797 | *n/s* | 2.40E-04 | 1.25E-03 |
|  |  |  |  |  |  |  |
|  |  |  |  |  |  |  |
| CP37 | Frequency (%) | | | p-value | | |
| TCR Sequence | Unstimulated (n=4,949) | SL9 (n=14,939) | SL9CR-2 (n=9,414) | Unstimulated vs SL9 | Unstimulated vs SL9CR-2 | SL9 vs  SL9CR-2 |
| CASRPTGATPYGYTF | 0.181855 | 0.287837 | 0.605481 | *n/s* | 1.37E-04 | 2.81E-04 |
| CASRRGTGELFF | 0.080824 | 0.348082 | 0.499256 | 6.17E-04 | 1.06E-05 | *n/s* |
| CASSFGTPYGYTF | 0 | 0.087021 | 0.159337 | *n/s* | 1.95E-03 | *n/s* |
| CASSLDSEQYF | 96.3225 | 89.9458 | 89.6750 | 1.05E-11 | 2.47E-22 | 7.61E-06 |
| CASSLSLGWSEQYF | 0.141443 | 0.368164 | 0.478011 | *n/s* | 6.67E-04 | *n/s* |
| CASSQGQPSTDTQYF | 0.040412 | 0.220898 | 0.308052 | *n/s* | 2.62E-04 | *n/s* |
| CASSTTGIGDTGELFF | 0.181855 | 0.207511 | 0.499256 | *n/s* | 1.86E-03 | 1.61E-04 |
|  |  |  |  |  |  |  |
|  |  |  |  |  |  |  |
| CP41 | Frequency (%) | | | p-value | | |
| TCR Sequence | Unstimulated (n=742) | SL9 (n=3,363) | SL9CR-2 (n=3,301) | Unstimulated vs SL9 | Unstimulated vs SL9CR-2 | SL9 vs  SL9CR-2 |
| CASSFDHEQFF | 0.943396 | 1.30836 | 0.545289 | *n/s* | *n/s* | 2.49E-04 |
| CASSMYGVDTEAFF | 0 | 1.27862 | 6.30112 | *n/s* | 1.13E-16 | 6.44E-27 |

* identical TCR sequence by AA but differs by nucleic acid sequence.

*n/s* indicates no significant difference or too few cells to make comparison
