# Supplementary material for: Cross-reactive microbial peptides can modulate HIV-specific CD8+ T cell responses
Source: PLoS One. 2018 Feb 21;13(2):e0192098. doi: 10.1371/journal.pone.0192098 (PMC5821448; doi:10.1371/journal.pone.0192098)
Supplement: S3 Table — (DOCX) [file pone.0192098.s003.docx]

**S3 Table. Individual p-values from PBMC suppression assay**

|  | CEF vs | | | | | | | | | |
| --- | --- | --- | --- | --- | --- | --- | --- | --- | --- | --- |
|  | KK10 | KK10CR-1 | KK10CR-2 | KK10CR-3 | KK10CR-4 | KK10CR-5 | KK10CR-6 | KK10CR-7 | KK10CR-8 | KK10CR-9 |
| CP2A | **0.0094** | 0.9999 | **0.0463** | 0.9166 | 0.9999 | 0.9999 | 0.9999 | 0.9238 | 0.9703 | 0.9999 |
| CP3A | **0.0001** | 0.7455 | **0.0006** | 0.7455 | 0.7455 | 0.7455 | 0.7844 | **0.0002** | **0.0004** | 0.7455 |
| CP4A* | 0.0585 | 0.9999 | 0.2274 | 0.9999 | 0.9999 | 0.9999 | 0.9999 | 0.9999 | 0.9999 | 0.9999 |
| ES31 | **0.0001** | 0.9694 | **0.0001** | 0.9694 | 0.9999 | 0.9999 | **0.0025** | 0.9694 | **0.0005** | 0.9999 |
| ES9 | **0.0001** | 0.9057 | 0.9057 | 0.9057 | 0.9057 | 0.9057 | 0.9057 | **0.0001** | **0.0001** | 0.9057 |

|  | CEF vs | | | | | | | | | |
| --- | --- | --- | --- | --- | --- | --- | --- | --- | --- | --- |
|  | SL9 | SL9CR-1 | SL9CR-2 | SL9CR -3 | SL9CR -4 | SL9CR -5 | SL9CR -6 | SL9CR -7 | SL9CR -8 | SL9CR -9 |
| CP2A | **0.0017** | **0.0001** | 0.9798 | 0.7369 | 0.9999 | 0.9999 | 0.9999 | 0.9999 | 0.9999 | 0.9999 |
| CP37 | **0.0001** | 0.4063 | **0.0001** | 0.9999 | 0.9999 | 0.873 | **0.0001** | 0.9999 | 0.9999 | 0.4063 |
| CP41 | **0.0001** | **0.0015** | **0.0001** | 0.9941 | 0.9941 | **0.0038** | 0.9941 | 0.9941 | 0.9941 | 0.1044 |
| CP42* | 0.984 | 0.6053 | 0.0508 | 0.984 | 0.6902 | 0.984 | 0.984 | 0.984 | 0.984 | 0.984 |
| CP43* | n/a | n/a | n/a | n/a | n/a | n/a | n/a | n/a | n/a | n/a |
| CP44* | n/a | n/a | n/a | n/a | n/a | n/a | n/a | n/a | n/a | n/a |
| CP12 | **0.0005** | 0.9999 | 0.4523 | 0.9999 | 0.7533 | 0.3134 | 0.9998 | 0.9999 | 0.9999 | 0.9999 |

Calculated by ANOVA with p-values adjusted using Holm-Sidak’s multiple comparisons test.

* indicates donor that did not show suppression of infection above limit of quantification after stimulation with wild type HIV peptide

n/a indicates comparisons where p-values are impossible to measure due to all values = 0.
